# Critical Parameters of the *In Vitro* Method of Vascular Smooth Muscle Cell Calcification

**DOI:** 10.1371/journal.pone.0141751

**Published:** 2015-11-10

**Authors:** Luis Hortells, Cecilia Sosa, Ángel Millán, Víctor Sorribas

**Affiliations:** 1 Department of Toxicology, University of Zaragoza, Veterinary Faculty, Zaragoza, Spain; 2 Institute of Materials Science of Aragón, CSIC – Universidad de Zaragoza, Zaragoza, Spain; Brigham and Women's Hospital, Harvard Medical School, UNITED STATES

## Abstract

**Background:**

Vascular calcification (VC) is primarily studied using cultures of vascular smooth muscle cells. However, the use of very different protocols and extreme conditions can provide findings unrelated to VC. In this work we aimed to determine the critical experimental parameters that affect calcification *in vitro* and to determine the relevance to calcification *in vivo*.

**Experimental Procedures and Results:**

Rat VSMC calcification *in vitro* was studied using different concentrations of fetal calf serum, calcium, and phosphate, in different types of culture media, and using various volumes and rates of change. The bicarbonate content of the media critically affected pH and resulted in supersaturation, depending on the concentration of Ca^2+^ and Pi. Such supersaturation is a consequence of the high dependence of bicarbonate buffers on CO_2_ vapor pressure and bicarbonate concentration at pHs above 7.40. Such buffer systems cause considerable pH variations as a result of minor experimental changes. The variations are more critical for DMEM and are negligible when the bicarbonate concentration is reduced to ¼. Particle nucleation and growth were observed by dynamic light scattering and electron microscopy. Using 2mM Pi, particles of ~200nm were observed at 24 hours in MEM and at 1 hour in DMEM. These nuclei grew over time, were deposited in the cells, and caused osteogene expression or cell death, depending on the precipitation rate. TEM observations showed that the initial precipitate was amorphous calcium phosphate (ACP), which converts into hydroxyapatite over time. In blood, the scenario is different, because supersaturation is avoided by a tightly controlled pH of 7.4, which prevents the formation of PO_4_^3-^-containing ACP.

**Conclusions:**

The precipitation of ACP *in vitro* is unrelated to VC *in vivo*. The model needs to be refined through controlled pH and the use of additional procalcifying agents other than Pi in order to reproduce calcium phosphate deposition *in vivo*.

## Introduction

Medial vascular calcification (MVC) is a degenerative process inherent in some congenital disorders, and it is also common in type 2 diabetes, chronic kidney disease (CKD), and the elderly [1,2]. MVC impairs cardiovascular hemodynamics, therefore increasing morbidity and mortality. In the case of CKD, additional hyperphosphatemia (complicated by secondary hyperparathyroidism and uremic toxins) has been described as an independent risk factor for the development of MVC [3].

The pathogenesis of this ectopic calcification has been intensely studied over the last fifteen years, and the results of studies show the occurrence of critical phenomena such as the *trans*differentiation of vascular smooth muscle cells (VSMC) into osteoblast-like cells, apoptosis, and the formation of matrix vesicles and apoptotic bodies that increase nanocrystal deposition, among other phenomena [1,2,4–6]. Yet many questions remain unanswered, including the key initiating causes, contributing factors, the order of events, etc.

The pathogenesis of MVC has been studied using both *in vitro* and *in vivo* models. *In vitro* models encompass the use of aortic rings [7], intact vessels (i.e., *ex vivo*) [8], and mainly cultures of VSMC from different donor species [9,10]. Unfortunately, there are serious deficiencies in the VSMC culture model, such as the lack of extracellular elastin fibers (which seem to constitute the main mineral deposition site in arteries) [11] or uncertainty about the phenotype of the cells in a culture, ranging from the differentiated (*contractile*) to the proliferative (*synthetic*) states [12].

Nevertheless, VSMC cultures have been used to study the relevance of apoptosis, nanoparticle endocytosis, the synthesis of calcification inhibitors, exosome release, and so on. Moreover, in the specific case of CKD associated with hyperphosphatemia, one seminal work showed that a VSMC culture with Pi concentrations comparable to those seen in hemodialysis patients (> 1.4mM) increased the mineral deposition in cultures and the expression of the osteoblastic markers osteocalcin and Runx2 [13]. Since then, many groups, including ours, have been using this model of VC, but with very different conditions pertaining to culture media composition, Pi concentration, etc. The variability of the experimental conditions can be highly critical, because slight differences in some parameters can result in very dissimilar outcomes. For example, ionic concentrations that are harmless *in vivo* can lead to massive precipitation *in vitro*, therefore leading to invalid conclusions. In other cases, Pi has been used at concentrations that are never found *in vivo* or are even incompatible with life.

In this work we have analyzed several parameters that affect VSMC calcification *in vitro*, including the concentrations of calcium, phosphate, bicarbonate, pH, and the culture medium, thereby showing the major dependence between the assay and the physicochemical conditions. We also go over the process of calcium phosphate precipitation, with special attention to the precipitating phase, phase transformations, supersaturation of the amorphous and crystalline calcium phosphate phases, spontaneous nucleation, induction times, and precipitation *in vitro*.

## Materials and Methods

### Cell culture

VSMC were obtained from the aorta of 2-month-old male Wistar rats (Janvier SAS, Berthevin, France) by manual dissection and collagenase digestion as described [6,14]. This was performed in accordance with European legislation and was approved by the Ethical Committee of the University of Zaragoza. Rats were maintained with free access to food and water, and after deep anesthesia with pentobarbital, aortas were obtained and immediately processed. VSMC were grown in Minimum Essential Medium with 10% fetal calf serum, glutamine, and antibiotics, between passages 4 to 10, and in a 5% CO_2_ atmosphere using an automatic CO_2_ incubator (Barnstead International, model 490-1CE, Dubuque, IA, USA). When the cells reached 90% confluence, they were made quiescent using 0.2% FCS-containing medium. All culture media and reagents were from Gibco (Paisley, UK), with the exception of some confirmatory experiments described in Results, which were also conducted using identical MEM and DMEM from Sigma-Aldrich (St Louis, MO) and from Lonza (Basel, Switzerland).

### Calcification assays

For the calcification assays, VSMC were grown in multi-well plates and chamber slides. Cells were incubated with different media depending on the experiment: Minimum Essential Medium (MEM, 26.19mM bicarbonate), MEM plus 18mM extra bicarbonate (final concentration of 44.05mM), or Dulbecco’s Modified Eagle Medium (DMEM, 44.05mM bicarbonate). In some experiments, DMEM:F12 was used instead, containing 14.29mM sodium bicarbonate and 15.01mM 4-(2-hydroxyethyl)-1-piperazineethanesulfonic acid (HEPES). Several Pi concentrations were used from a stock of 100mM KH_2_PO_4_/K_2_HPO4, pH 7.4. The deposited calcium was quantified using a colorimetric Bioassay Systems kit (Hayward, CA) after overnight solubilization of the deposits with 0.6 N HCl [6,15]. Calcium was expressed per milligram per surface unit.

### pH determinations

The pH of the culture medium was determined with a pH & Ion-Meter GLP 22, using a pH microelectrode 5208 (Crison, Barcelona, Spain). pH changes were monitored continuously, either in a laminar flow hood or inside a CO_2_ incubator after temperature stabilization to 37°C. The accuracy of the determinations was checked using the pH calibrators (Crison) at both room temperature and at 37°C.

### Biochemical assays

Cell death was evaluated colorimetrically by the activity of the lactate dehydrogenase (LDH) released in the media, using a commercial kit (Roche, Mannheim, Germany). LDH activity was expressed as a percentage of maximal reaction, which was obtained through the lysis of all cells using Triton X-100, as described [15]. Cell death was confirmed by microscopy after staining the cells with acridine orange/ethidium bromide fluorescent dyes, as described [15]. Live cells are stained green by acridine orange, while ethidium bromide only stains dead cells in red, because it is not accumulated in live cells.

Alkaline phosphatase activity was determined in VSMC lysates using the traditional colorimetric assay of p-nitrophenyl phosphate, exactly as described, but for cell lysates [16]. VSMC were lysed with ice-cold radioimmunoprecipitation assay (RIPA) buffer (10mM Tris, pH 7.4; 2.5mM EDTA; 50mM NaF; 1mM Na_4_P_2_O_7_; 1% Triton X-100; 10% glycerol; 1% deoxycholate; 1 mg/mL aprotinin; 0.18 mg/mL phenylmethanesulphonylfluoride; 0.18 mg/mL orthovanadate; and 1% Triton X-100 in 0.9% NaCl), and after 5 minutes of centrifugation at maximal speed, the supernatant was processed. All reagents were obtained separately from Sigma.

For DNA and protein extractions, a GenElute Mammalian Genomic DNA Kit (Sigma Aldrich) and a similar RIPA buffer (50 mM Tris, pH 7.5; 150 mM NaCl; 0.1% SDS; 0.5% deoxycholate; 1% Triton X-100; and protease inhibitor cocktail, Sigma) were used, respectively. For protein quantitation, a BCA kit (Pierce BCA Protein Assay, Thermo Scientific, Rockford, IL) was also used. The Pi and calcium contents of the culture media were determined with the corresponding colorimetric kits from Bioassay Systems.

### Real-time PCR

For retrotranscription, a Transcriptor First Strand cDNA Synthesis Kit was used, and for amplification, a LightCycler FastStart Master SYBR Green I kit was used (both from Roche Applied Science, Mannheim, Germany).

The relative expressions of Runx2, Msx2, and tissue non-specific alkaline phosphatase (TNAP) RNAs were determined by real-time PCR using SYBR Green on a LightCycler 1.5 (Roche Applied Science, Mannheim, Germany), as described [15]. Glyceraldehyde 3-phosphate dehydrogenase (GAPDH) was used as a reference, in addition to a calibrator cDNA (retrotranscribed from pooled RNAs of VSMC incubated with either 1 or 2mM Pi in MEM for 10 days and combined in a single RNA population). The sequences of the primers were the following. GAPDH: Sense, TCCAGTATGACTCTACCCACG, antisense, CACGACATACTCAGCACCAG. Runx2: Sense, CTGCCGAGCTACGAAATGCC, antisense, GGCCACTTGGGGAGGATTTG. Msx2: Sense, ACCGAAGGGCTAAGGCAAAA; antisense, CGCTGTATATGGATGCCGCT. TNAP: Sense, CAGAGAAAGAGAAAGACCCCAG; antisense, CTGTCACTGTGGAGACGC.

#### Crystal study

The formation and size of calcium phosphate particles were examined by dynamic light scattering, DLS, using a Nanosizer ZS (Malvern, Worcestershire, UK). Particles were measured at different incubation times of the culture media in the 5% CO_2_ incubator, using the indicated concentrations of phosphate.

The ultrastructure and chemical composition of calcium phosphate deposits were determined as described [17] using a transmission electron microscope (TEM, JEOL 2000FXII) equipped with an INCA-200 X-Sight microanalysis system (Oxford Instruments). Samples were prepared by dispersing ground powders from the bottom of the culture wells in hexane, and the dispersion was evaporated on a carbon-coated microscope grid.

### Statistical analysis

Each experiment was repeated at least twice, and the quantitative experiments at least three times, run in triplicate. For statistics and non-linear regressions, Prism 5.0 software (GraphPad Software Inc., La Jolla, CA) was used. Significant differences were determined using a one-way analysis of variance (ANOVA) with Tukey’s multiple comparison test. For the comparison of two means and for some confirmatory purposes, a t-test was also used in some cases, as indicated, and the difference was considered significant when p < 0.05. The P values are indicated in the figure legends according to the type of analysis used.

## Results

### VSMC death as a single cytotoxicity end point

**Fig 1:**
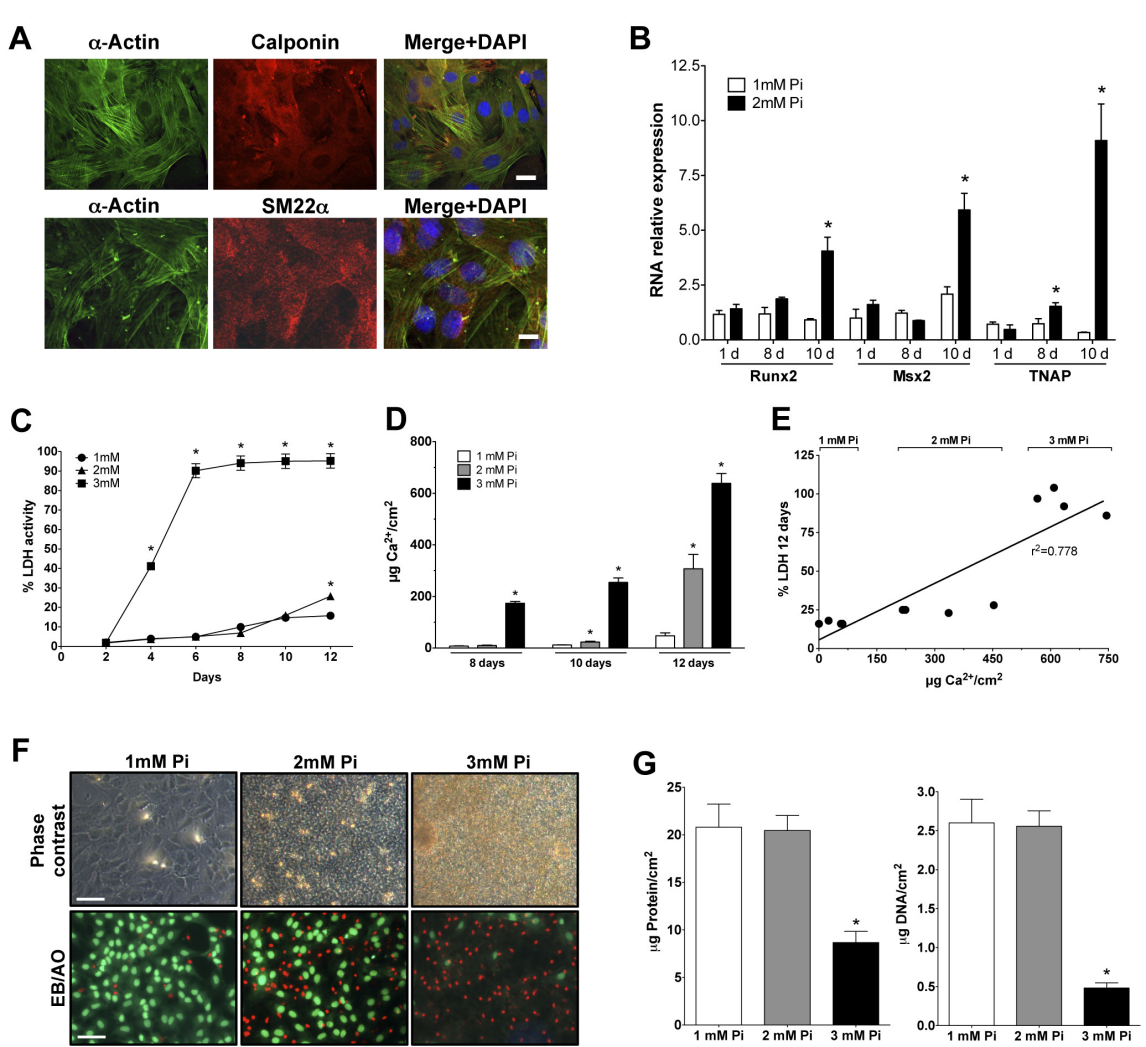
Cell death during in vitro calcification. (A) Microphotographs of rat VSMC at passage 3, immunodecorated with smooth muscle markers. Bar, 20μm. (B) Osteogene RNA expression in VSMC cultured in MEM containing 1 or 2mM Pi for 1, 8, or 10 days. ANOVA p < 0.0001 for all three genes. *Significantly different from the corresponding 1mM Pi with Tukey’s and a t-test. (C) LDH activity, as a percentage of day 0, in VSMC incubated using Pi concentrations for up to 12 days in MEM. ANOVA p < 0.0001, *Different from the corresponding 1mM Pi with Tukey’s test. At 12 days, the difference at 2 and 1mM Pi is also significant with a t-test (p < 0.0001). (D) Calcium deposition at days 8, 10, and 12. ANOVA, p < 0.0001; *Different from the corresponding 1mM Pi with Tukey’s test. (E) Calcium deposition and LDH activity correlation after 12 days of calcification *in vitro*. (F) Phase contrast and EB/AO staining microphotographs after 12 days. Bar, 50μm. (G) VSMC, total protein (ANOVA, p < 0.005), and DNA content (ANOVA p < 0.005) after 12 days of Pi treatments; *Different from 1mM with Tukey’s test.

To check that our cultures were VSMC and behaved as such, we stained them with antibodies against the smooth muscle markers α-actin, calponin, and Sm22α, evidencing that practically all cells were VSMC (Fig 1A). The cultures also calcified and expressed bone-related genes. RNA was extracted from cells after one, eight, and ten days of treatment with 1 or 2mM Pi in MEM. Runx2, Msx2, and TNAP RNA expressions were minimal, and the differences were non-significant at 24 hours (Fig 1B). After 8 days of incubation with 2mM Pi, only the TNAP expression increased significantly above the 1mM Pi level. However, strong and significant increases of expression were observed in Runx2, Msx2, and TNAP RNAs after 10 days of incubation with 2mM Pi in MEM.

Then, VSMC survival during calcification was analyzed by correlating total cell death and calcium deposition. Quiescent VSMC grown in 24-well plates with 0.2% FCS in MEM were treated with 1, 2, or 3mM Pi for 12 days. LDH activity was determined at every culture medium change (every two days) and is shown as accumulated activity (Fig 1C). This revealed that VSMC death increased as the Pi concentration increased, thereby increasing the calcium deposition (Fig 1D), at a correlated rate with LDH activity (r^2^ = 0.7778, Fig 1E). In fact, LDH significantly increased as soon as apparent calcification was observed by phase contrast microscopy of the cells (e.g., Fig 1E). Exposure time to precipitated calcium is also important for cell survival, as shown by the different LDH percentages between 3mM Pi for 10 days and 2mM Pi at day 12 (Fig 1C): while in both cases the calcium content of the deposits is similar (Fig 1D), VSMC were only exposed to the calcium deposits of the 2mM Pi condition for approximately one day (after 10 days the deposits were minimal), while in the 3mM Pi condition, deposits were already massive after at least 8 days. Cell death results were corroborated by staining VSMC with EB/AO at the 12^th^ day of treatment (Fig 1F). Similarly, total VSMC protein and the DNA content of the calcifying wells decreased during Pi-induced calcification (Fig 1G), a finding that should be kept in mind when calcification is expressed as calcium per milligram of protein to avoid the artificial magnification of deposits.

### Effect of several cell culture parameters on VSMC viability

**Fig 2:**
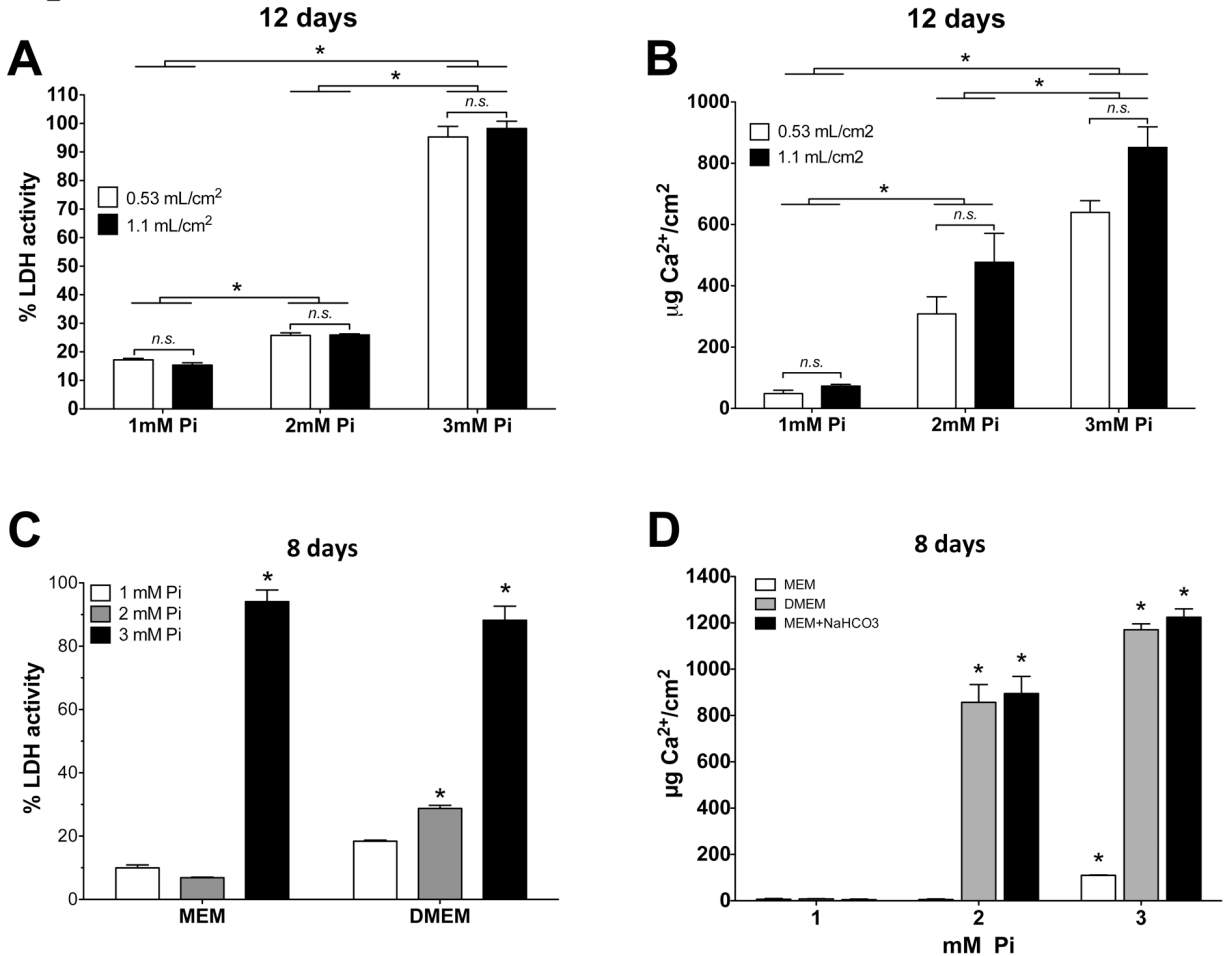
Effect of culture parameters on Pi cytotoxicity in VSMC. (A) Effect of the indicated MEM volume and Pi concentrations on cell viability. ANOVA, p < 0.0001; *Different from the corresponding 1mM with Tukey’s test. For simplicity, asterisks show the comparisons of single means after combining the values from the different media volumes at the same Pi concentration. *n*.*s*.: non-significant differences caused by media volumes. (B) Calcium deposition in the same experiment as A. Statistical analysis as in A. (C) Effect of culture media type on Pi cytotoxicity after 8 days. MEM and DMEM ANOVA are p < 0.0001; *Different from the corresponding 1mM with Tukey’s test. (D) Quantification of calcium in deposits after 8 days of Pi treatments, using either MEM or DMEM, or MEM plus a bicarbonate concentration as for DMEM. All three ANOVAs (one per culture media) are p < 0.0001; *Different from the corresponding 1mM with Tukey’s test.

Several culture parameters that could affect cell viability were tested. It was determined that viability was not affected for 12 days either by an FCS percentage at 0–0.6% for quiescence in MEM or by renewing the medium every 2 vs. 6 days (not shown). To test for the effect by culture media volume on cell viability, cells were incubated during 12 days in MEM with 1, 2, or 3mM Pi, using either 0.53 or 1.1 mL/cm^2^ (i.e., 1 or 2ml per well in a 24-well plate), with media changes every 2 days. LDH determination (Fig 2A) and EB/AO staining (not shown) revealed no effect by the culture media volume. In the same experiment, the trend of the calcium content determination in the deposits was similar to that of LDH (Fig 2B). In this case, however, while a Tukey’s multiple comparison post-test did not find a significance of differences between the means of 3mM Pi using 0.53 or 1.1 mL/cm^2^ (p > 0.05) regarding calcium depositions, a t-test found a minimal significance with a p value of 0.0339.

However, the type of culture media had a major effect on cell viability and calcium deposition. VSMC were incubated for 8 days with MEM or DMEM containing 1, 2, or 3mM Pi, 0.2% FCS, and 0.53 mL/cm^2^, which was renewed every 2 days. LDH activity was similar with both media at 3mM Pi, but at 2mM Pi, only cells in DMEM showed a significant increase in cell death (Fig 2C). The effect on calcium deposition was similar. At 2mM Pi, DMEM accumulated 150 times more calcium than MEM, while at 3mM Pi, the calcium content was only 10.6 times higher in DMEM, considering that calcium deposition was already significant in MEM (Fig 2D). The fact that the differences in the formation of deposits were always greater than the differences in LDH activity (e.g., by comparing Fig 2B and 2D with Fig 2A and 2C) suggests that deposition precedes and causes cell death. Nevertheless, the effect of nanoparticles in suspension (see below) cannot be excluded as a cause of cell death. Dead cells, in turn, will serve as new nucleation sites, which will further increase the deposition of calcium phosphates.

The most relevant difference between MEM and DMEM is the bicarbonate content: 26.19 vs. 44.05mM, respectively. When we used MEM supplemented with 18mM NaHCO_3_ to achieve the same concentration as in DMEM, the calcium content in deposits was similar to the values obtained with DMEM, using either 2 or 3mM Pi (Fig 2D).

### Effects on culture medium pH during calcification assays

**Fig 3:**
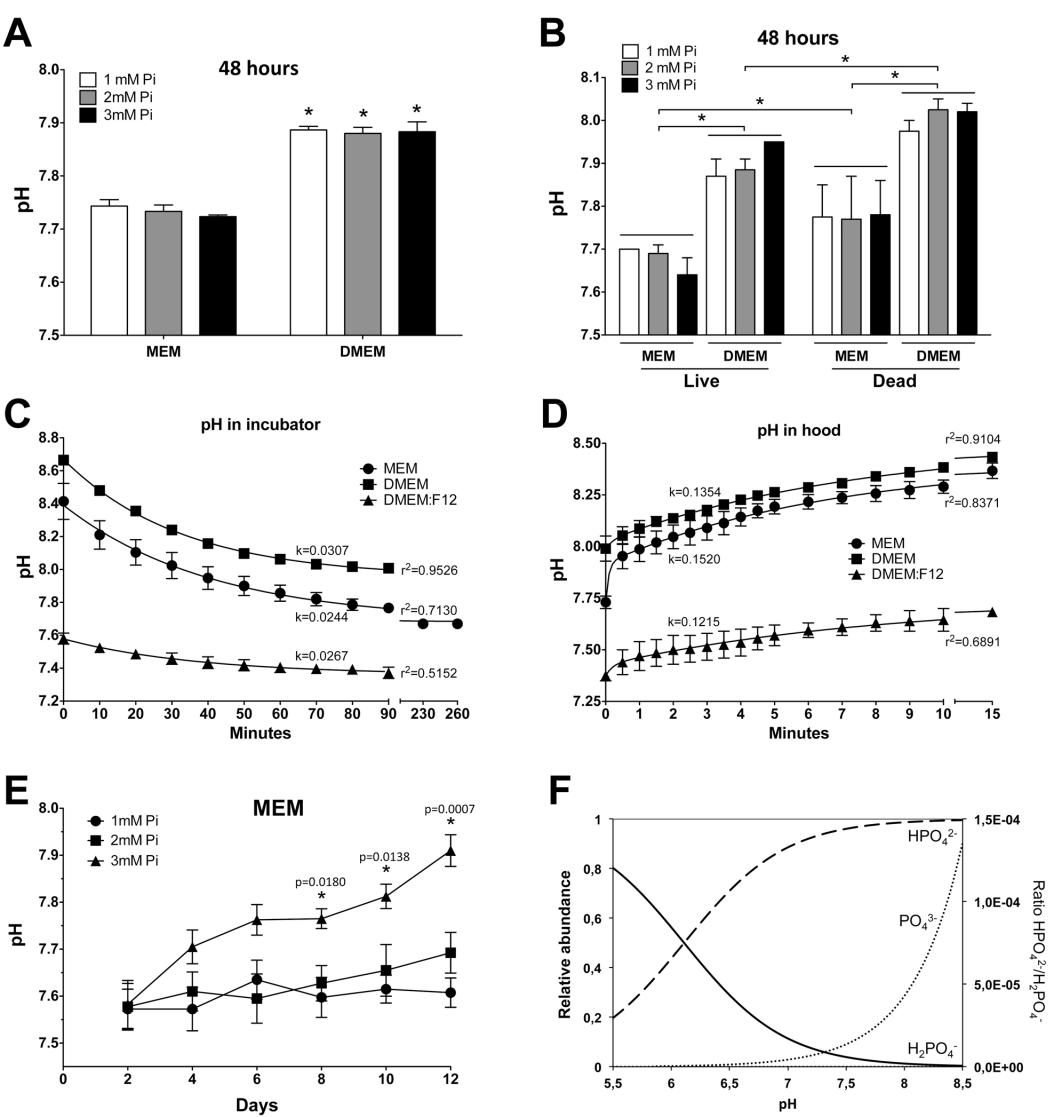
pH of culture media. (A) pH of culture media determined in a laminar flow hood after 2 days of Pi treatments. ANOVA, p < 0.0001. *Different from the corresponding Pi concentration in MEM with Tukey’s test. (B) pH of media after two days of Pi treatments on live and dead VSMC. ANOVA, p < 0.0001 for the four groups. Because there were no differences of pH values due to the Pi concentrations within each group, multiple comparisons were made using the means of each subgroup. All comparisons are significantly different with Tukey’s test. (C) Evolution of the pH of newly made media in 24-well plates inside a 5% CO_2_ incubator. The experimental pH values were fitted to an exponential decay curve, *pH*_*t*_ = (*pH*_0_ − *pH*_∞_) × *e* ^−*k*∙*x*^ + *pH*_∞_, by nonlinear regression, where pH_0_ is the pH at time 0 (in the hood) and pH∞ is the lowest pH reached, i.e., the plateau. Constant rates (k) and r^2^ are also shown for each fit. (D) Evolution of pH in the hood when cultures were moved from the 5% CO_2_ incubator. After 0.5 minutes of rapid rise, the pH rose more slowly, and therefore the data were fitted to a two-phase exponential equation:
$${pH}_{t}={pH}_{\infty }+({pH}_{0}-{pH}_{\infty })\times {\%}_{Fast}\times 0.1\times e^{-Kfast∙x}+({pH}_{0}-{pH}_{\infty })\times (100-{\%}_{Fast})\times 0.1\times e^{-Kslow∙x}.$$
The constant rates in the figure refer to the slow (long) phase, while the r^2^ corresponds to the whole fit. (E) Evolution of pH in MEM containing 1–3mM Pi during the calcification of VSMC for 12 days. Measurements were made inside a 5% CO_2_ incubator. An ANOVA was conducted for each determination day. *Different from the corresponding 1mM Pi pH value. (F) Variation of the relative concentration of different phosphate species in solution according to the pH. The PO_4_^3-^ ratio corresponds to the right vertical axis.

Because bicarbonate is the main buffer in a CO_2_ incubator, the pH of the culture media was measured. Fig 3A shows that after two days of incubation, the determination of pH (outside the incubator, in the hood) revealed that the pH of DMEM was approximately 0.2 pH units higher than that of MEM at all three Pi concentrations. To check the effect by cell metabolism on this pH difference, live and dead VSMC (previously fixed with 2% paraformaldehyde) [18] were incubated for 48 hours with MEM or DMEM and at the three concentrations of Pi (Fig 3B). The pH of the media was determined, thereby revealing that it was not significantly altered by any Pi concentration, but both DMEM and cell death together increased the pH: the lowest pH was observed in live cells with MEM, and the highest pH (around 8.0) was observed in dead cells with DMEM. These findings strongly suggest that cell metabolism is preventing the rise of pH, although the pH values were higher than expected under all conditions. Because a high pH can affect the equilibrium of phosphate and calcium ions in a solution and in the precipitation process, therefore explaining the differences in calcium deposition, the changes in the pH of the culture media were studied in greater detail. We measured the pH of the media during the various major steps of calcification *in vitro*. First, the pH was determined in MEM, DMEM, and DMEM:F12 (which contains 14.29mM NaHCO_3_ and 15.01mM HEPES). After the various media were prepared at 37°C in the hood, the pH was measured (time 0). The media were then distributed in a 24-well plate at 1ml per well, and the evolution of the pH was measured inside the CO_2_ incubator (Fig 3C). This experiment revealed that the pH of the three media dropped exponentially in the 5% CO_2_ atmosphere. The data were fitted to a one-phase, exponential decay equation by non-linear regression (see figure legend), thereby confirming the experimental findings: at time 0 (in the hood, pH_0_), the pH values of DMEM, MEM, and DMEM:F12 were 8.67 ± 0.02, 8.39 ± 0.05, and 7.58 ± 0.03, respectively. The lowest pH values reached at equilibrium were, respectively, 7.95 ± 0.03, 7.68 ± 0.06, and 7.36 ± 0.04, meaning a drop of 0.72 ± 0.03, 0.71 ± 0.07, and 0.22 ± 0.04 pH units, with half times of 22.6, 28.4, and 26.0 minutes, also respectively. A global fit revealed that the curves were statistically different (p < 0.0001, F = 265.0). When the cultures were moved in the reverse direction, i.e., from the CO_2_ in the incubator (at a stable pH) to the hood, the pH increased in two steps, especially in the case of MEM (Fig 3D). The pH rose quickly during the first 30 seconds and then continued to rise more slowly until equilibrium, therefore fitting a two-phase exponential curve. The pH values at the plateau were 8.50 ± 0.05, 8.41 ± 0.08, and 7.74± 0.10 for DMEM, MEM, and DMEM:F12, respectively, and these values were reached at half times (of the slow phase) of 5.1, 4.6, and 5.7 minutes, also respectively. Again, a global fit showed that the curves were statistically different (p < 0.0001, F = 295.8). These experiments, which were performed using media from Gibco, were confirmed with identical results using equivalent media from Sigma-Aldrich and from Lonza (not shown). The results show that neither MEM or DMEM are suitable buffers for VSMC in a 5% CO2 atmosphere, because they do not reach the desired pH 7.4, which is, however, obtained with DMEM:F12 containing low bicarbonate and HEPES.

Based on the aforementioned differences in pH according to the concentrations of CO_2_ and bicarbonate and based on the fact that the pH was higher with dead cells (Fig 3B), we measured the pH of MEM during the calcification process and inside the CO_2_ incubator using 1–3mM Pi in MEM (changed every two days with 0.53mL MEM/cm^2^). Fig 3E shows that the pH paralleled the rates of cell death and calcium deposition during calcification (Fig 1C and 1D), therefore increasing at 3mM Pi over time and increasing with the Pi concentration, with a slight rise at 2mM Pi after 12 days.

These findings strongly suggest that cell metabolism is preventing an even higher rise of pH and that the increased pH during calcification is mostly caused by the progressive loss of live cells rather than by the calcification conditions. Therefore, the pH of culture media increases during calcification as a consequence of cell death, which further increases the precipitation rate.

### Nucleation of calcium phosphates

**Fig 4:**
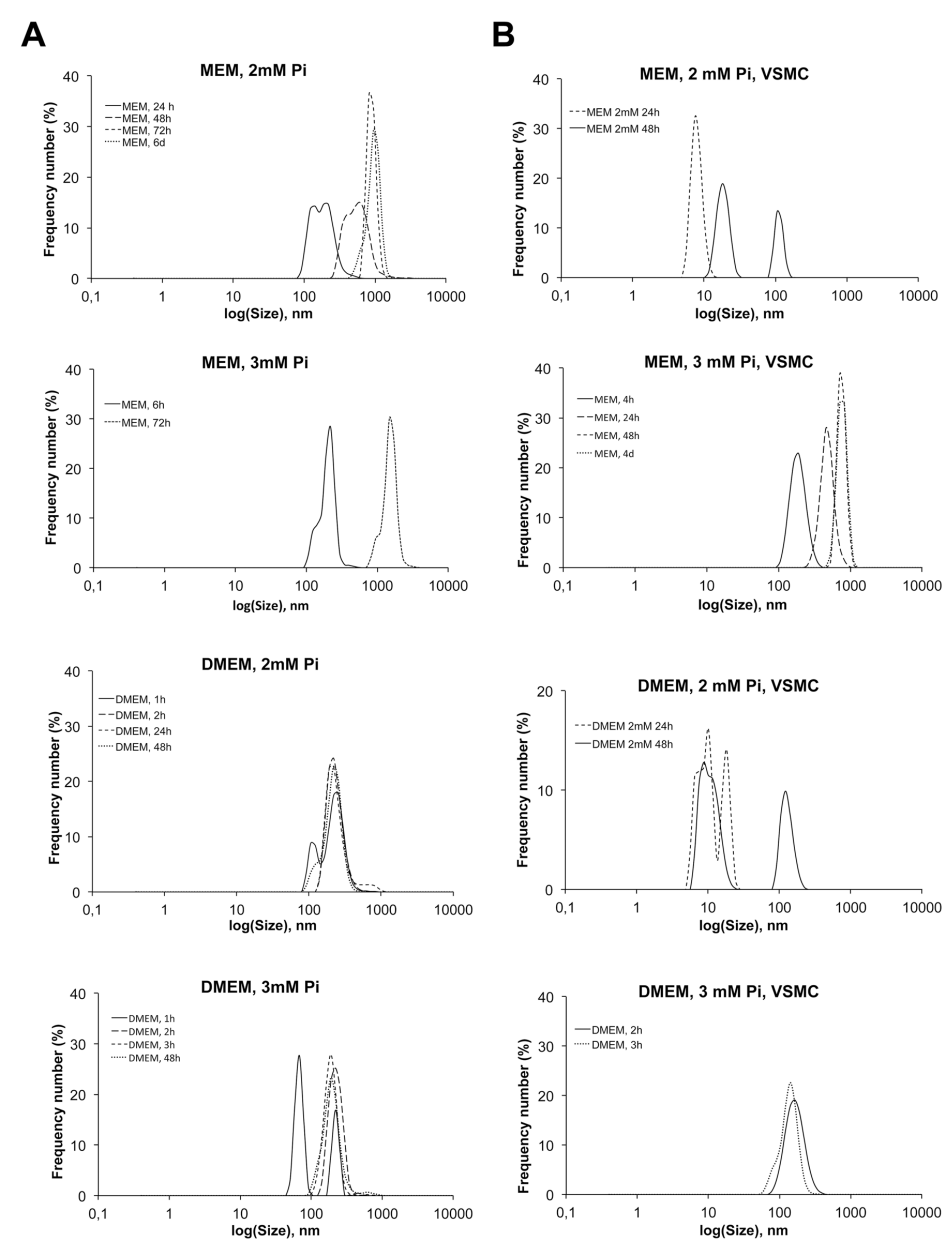
DLS analysis of the nanocrystal formation rate. (A) Graphs showing the formation of nuclei in MEM or DMEM without cells, in the CO_2_ incubator, at the indicated times, and in the presence of 2 or 3mM Pi. DLS determinations were made outside the CO_2_ incubator. (B) The same experiment as in A, in the presence of VSMC, with the indicated conditions of Pi concentration and incubation times.

The initiation of calcium phosphate precipitation *in vitro* was studied by DLS. First we studied nucleation in the media without cells: MEM ([HCO_3_^-^] = 26.19mM) and DMEM ([HCO_3_^-^] = 44.05mM) with different phosphate concentrations and constant calcium (Table 1). The solutions were maintained in culture dishes at 37°C in a CO_2_ incubator for up to 6 days (without changes), and aliquots were examined at several different moments in time by DLS to track the presence of calcium phosphate nuclei, in addition to the distribution and evolution of nuclei size. The solutions with a 1mM Pi content did not precipitate at any condition, even after 6 days. The corresponding DLS plots showed a peak at 10 nm at time 0, which did not evolve over the entire observation time. However, the DLS of MEM with 2mM Pi showed peaks after some time, with a size larger than 100 nm that may correspond to calcium phosphate. The first apparition of these nuclei was observed after 24 hours. The size distribution of early nuclei showed two maxima at approximately 120 nm and 190 nm (Fig 4A). Then, after 48 hours, the size distribution shifted to larger sizes, thereby indicating the growth of nuclei to form stable particles, and at 3 days, larger particles of 1 μm were already present, which may correspond to the growth of particles or to particle aggregation. The size distribution was monomodal and remained this way for at least three more days. In 3mM Pi-MEM, nucleation was observed at 6 hours, with a nearly monomodal distribution centered around 220 nm and a shoulder at lower sizes. After 48 hours, the most frequent size was larger than 1 μm due to aggregation, and some deposits on the bottom of the well were already visible to the naked eye. In DMEM, nuclei formation was already observed after 1 hour in 2mM Pi solutions, with a bimodal size distribution and maxima of around 100 nm and 150 nm, similar to the early nuclei in MEM solutions. One hour later, however, the distribution was already monomodal and remained with minimal changes for up to 24 hours, when deposits were already evident at the bottom of the well. At 3mM Pi in DMEM, the results were the same, and only the size distribution shifted to slightly lower values after 3 hours, which could be associated with the fact that the precipitate becomes denser over time, as described below in the TEM characterization section.

**Table 1:** Characteristics of solutions.

[Ca^2+^]_o_	[PO_4_^3-^]_o_	buffer	[HCO_3_]	*pH*_*0*_ *-pH*_*∞*_	*a*(Ca)_o_	*a*(PO_4_)_o_	*S*	*S*	*S*	(HAP)
mM	mM		mM		mM	mM	(ACP)	(DCPD)	(OCP)	(HAP)
**1.8**	1	DMEM	44.05	8.67-7.95	0.58	0.32	1.15-0.74	0.78-0.78	23-13	49-24
**1.8**	2	DMEM	44.05	8.67-7.95	0.58	0.64	1.40-0.90	1.06-1.05	31-17	58-28
**1.8**	3	DMEM	44.05	8.67-7.95	0.58	0.96	1.54-1.00	1.24-1.23	36-20	63-30
**1.8**	1	MEM	26.19	8.39-7.68	0.58	0.32	0.96-0.63	0.78-0.77	18-10	37-18
**1.8**	2	MEM	26.19	8.39-7.68	0.58	0.64	1.18-0.77	1.06-1.05	24-13	44-21
**1.8**	3	MEM	26.19	8.39-7.68	0.58	0.96	1.30-0.85	1.24-1.23	28-15	47-23
**1.05**	1	DMEM:F12	14.29	7.58-7.36	0.34	0.32	0.36-0.33	0.62-0.61	7-5	15-8
**1.05**	2	DMEM:F12	14.29	7.58-7.36	0.34	0.64	0.44-0.41	0.83-0.82	9-7	17-10
**1.05**	3	DMEM:F12	14.29	7.58-7.36	0.34	0.96	0.48-0.45	0.96-0.96	11-8	19-11

Total initial concentration of [Ca^2+^]_0_ = 1.8mM for DMEM and MEM and 1.05mM for DMEM:F12; activity coefficient from Davies equation [19], γ = 0.32.

Ionic activity product of amorphous calcium phosphate, IAP(ACP) = [*a*(Ca^2+^)][*a*(PO_4_^3-^)]^0.74^[*a*(H^+^)]^0.22^ and K_sp_(ACP) = 2.29 x 10^-11^.

Ionic activity product of dicalcium phosphate dihydrate, DCPD, IAP(DCPD) = [*a*(Ca2+)][*a*(H_2_PO_4_^-^)] and K_sp_(DCPD) = 2.4 x 10^-7^.

Ionic activity product of octacalcium phosphate, OCP, IAP(OCP) = [*a*(Ca^2+^)]^8^[*a*(H_2_PO_4_^-^)]^2^[*a*(PO_4_^-^)]^4^ and K_sp_(OCP) = 2.5 x 10^-99^.

Ionic activity product of hydroxyapatite, HAP, IAP(HAP) = [*a*(Ca^2+^)]^10^[*a*(PO_4_^3-^)]^6^[*a*(OH^-^)]^2^ and

K_sp_(HAP) = 5.5 x 10^-118^.

The supersaturation ratios were calculated for the pH values at time 0 (pH_0_) and in equilibrium with 5% CO_2_ (pH_∞_) in air, and they are defined as S=(IAPKsP)1υ, where ν is the sum of the exponent numbers in IAP expression.

The formation of nuclei was clearly inhibited in the presence of cells (Fig 4B). In this case, the media were changed every 2 days to reproduce the experimental operations, and therefore only the size distributions determined up to 48 hours could be compared with those of the media without cells. For both MEM and DMEM at 2mM Pi, nucleation was not observed in the presence of VSMC for periods of up to 24 hours. Only some maxima were observed at a size of 10 nm, thus indicating that nucleation was not very extensive, because this peak disappears progressively as nucleation advances. At 48 hours, a peak at 100 nm started to appear, which was associated with the nucleation of nanoparticles in the DLS measurements in the MEM and DMEM solutions, as stated above. When the Pi concentration was 3mM, nuclei appeared later than in the same media without cells, but the sizes and distribution were similar: particles were observed after 4 hours in MEM and 2 hours earlier in DMEM. In experiments with 2.5mM Pi, nucleation could be seen after 4 hours in MEM and after 2 hours in DMEM (not shown).

### Characterization of Pi cytotoxicity

VSMC death during an *in vitro* assay can be caused by many mechanisms, such as directly by Pi toxicity, indirectly through nuclei formation and endocytosis, physically by massive crystal deposition, by alkalinity, by calcium depletion upon precipitation, etc.

**Fig 5:**
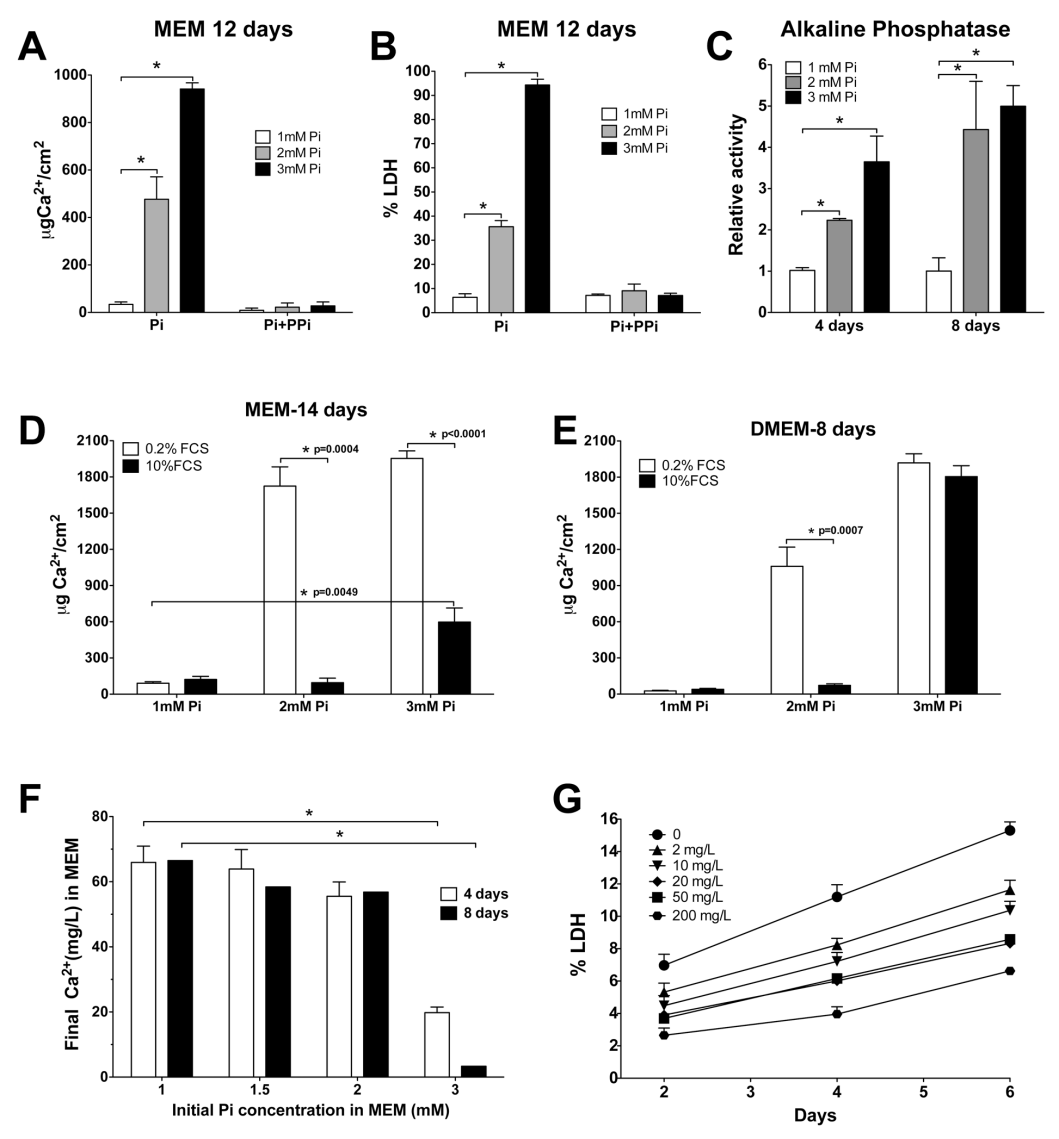
Mechanisms of cell death. (A) Prevention of calcification for 12 days with 12μM PPi in MEM. ANOVA, p < 0.0001 for calcification without PPi; *Significantly different from 1mM Pi with Tukey’s multiple comparison test. B. Prevention of Pi-induced cell death with PPi in MEM; statistics are as in A. (C) Alkaline phosphatase activity relative to the 1 mM Pi condition in cell lysates incubated for 4 or 8 days with 1, 2, or 3mM Pi in MEM. ANOVA at 4 days, p = 0.0065; ANOVA at 8 days, p = 0.0195. *Significantly different from 1mM Pi with Tukey’s multiple comparison test. (D) Treatment of VSMC with MEM containing 1, 2, or 3mM Pi and 0.2–10% FCS, for 14 days. Asterisks indicate significant differences of the means with a t-test, and p values are shown. (E) Treatment with DMEM as in A, for 8 days. Statistics as in C. (F) Variation of the calcium concentration in MEM after 4 and 8 days of incubation using the indicated Pi concentrations. *p < 0.0001 with t-tests. (G) Cell mortality after incubating VSMC with the indicated concentrations of calcium in lab-made MEM.

To distinguish between these mechanisms, several assays were performed. VSMC were incubated with several concentrations of Pi in MEM for 12 days, in the presence or absence of 12μM pyrophosphate (PPi), a major calcification inhibitor. As expected, not only did PPi completely prevent deposition, even at 3mM Pi during the entire period (Fig 5A), but it also prevented cell death caused by the calcification conditions (Fig 5B). The possibility of a direct Pi cytotoxic effect was therefore discarded, even at 3mM Pi.

PPi is hydrolyzed by alkaline phosphatase (AP) into two molecules of inorganic phosphate. To measure the AP activity in VSMC during calcification, we used cell lysates of VSMC incubated for 4 and 8 days with MEM containing 1, 2, or 3mM Pi (Fig 5C). We found that activity was increased by the two treatments with high Pi at 4 and 8 days, which concurs with the increase in RNA abundance (Fig 1B). An interpretation of these results could be that the newly expressed AP activity causes a decrease in the abundance of the calcification inhibitor PPi, and therefore extra PPi is necessary to prevent Pi-induced calcium deposition and cell death. However, homogeneous precipitation can appear just a few hours after media preparation and earlier than TNAP overexpression. Therefore, and because TNAP is a membrane-bound enzyme, the hydrolysis of PPi could have a determinant role in heterogeneous precipitation during later steps of calcification *in vitro*, rather than in the early homogeneous precipitation.

While viability was not affected by an FCS percentage at 0–0.6% (see above), we checked the inhibitory effect of FCS on calcification. We treated VSMC with MEM (for 14 days) or DMEM (for 8 days) containing 1, 2, or 3mM Pi and 0.2 or 10% FCS. In the case of MEM, 10% FCS completely prevented calcium deposition at 2mM Pi, while at 3mM Pi, deposition was only 30% of the calcium content using 0.2% FCS (Fig 5D). When DMEM was used for 8 days, 10% FCS prevented 95% of the deposition that was observed using 2mM Pi and 0.2% FCS, but it did not prevent deposition when 3mM Pi was used (Fig 5E).

Because Ca/Pi nucleation can cause a depletion of free calcium in media, we also measured the concentrations of both ions in MEM after 4 and 8 days of treatment with several concentrations of Pi. The findings revealed that calcium, while originally present at 1.8mM, almost disappeared when 3mM Pi was used in MEM for 8 days, but it only dropped to 56.8 mg/L in the 2mM Pi media (Fig 5F). Accordingly, we checked the effect by different concentrations of calcium on VSMC viability. Calcium-free MEM was prepared using components from the same supplier, and after 6 days of incubation with different calcium concentrations, LDH determinations every 2 days revealed an increased mortality rate that was inversely dependent upon the calcium concentration (Fig 5G). However, while this increase does contribute to total cell death, it does not justify the extensive mortality observed during calcification.

### Ultrastructural analysis of deposits

**Fig 6:**
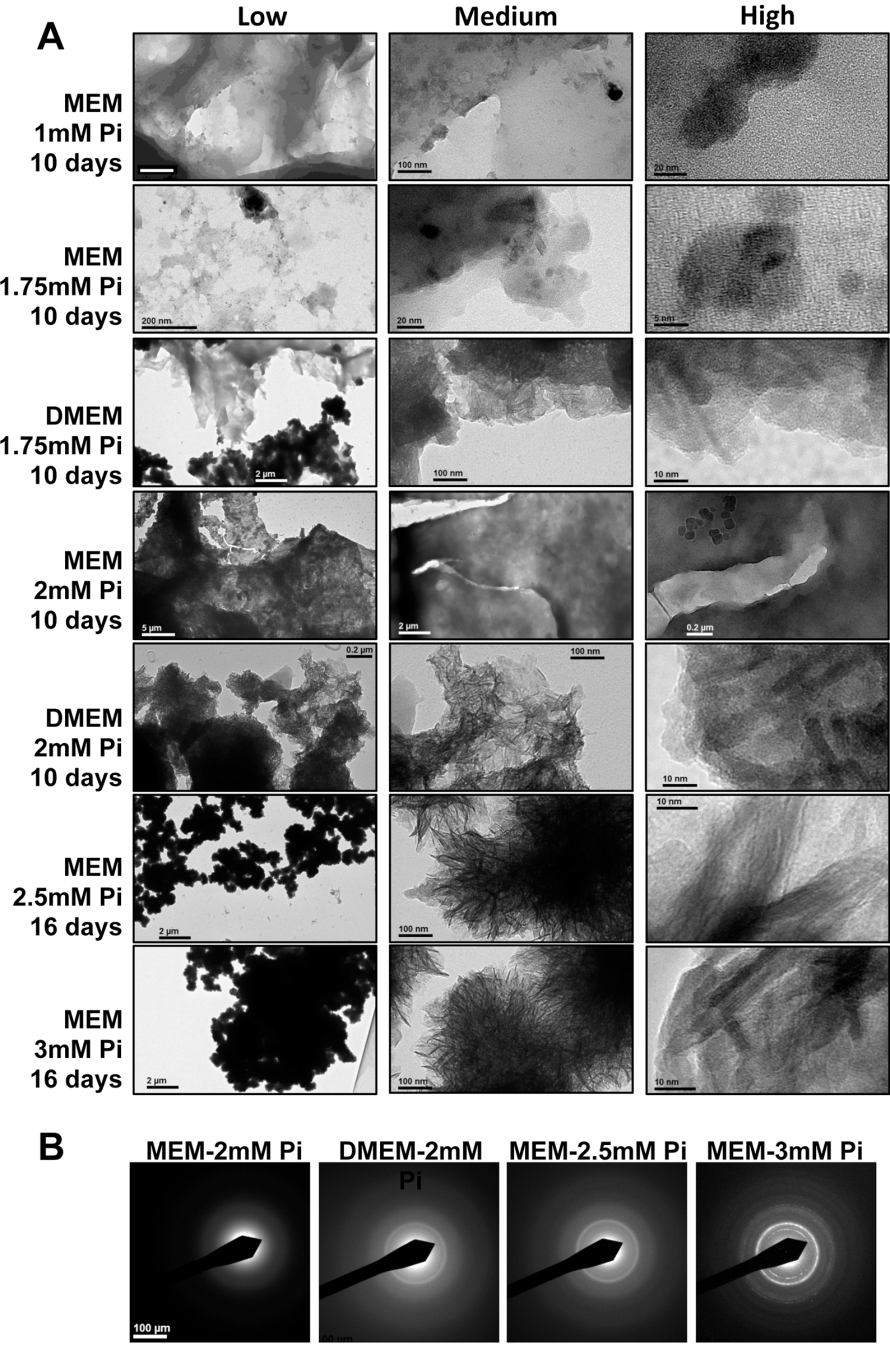
Ultrastructural analysis of deposits. (A) TEM images of deposits at different magnifications, from cells incubated as indicated at the left of each row. Images in the first column, at low magnification, are indicative of the density of the deposits, which is clearly increasing with the Pi concentration; images in the second column, with medium magnification, show the microstructures of the deposits, which gradually adopt a spherulite arrangement of needle-like particles as the concentration increases; images in the third column, at high magnification, show the shape and size of crystalline nanoparticles. (B) Electron diffraction patterns of cell culture deposits from A, showing an increasing crystallinity from MEM to DMEM, and from 2mM Pi to 3mM Pi. Using MEM at 2mM Pi for 10 days, ED revealed no reflections from crystal planes, however, when DMEM was used instead, crystallization rings at 2.81 and 3.52Å were observed, compatible with those of hydroxyapatite (2.82 and 3.44Å). MEM at 2.5 and 3mM Pi for 16 days also revealed a hydroxyapatite crystallization pattern, with clear reflections at 3mM.

The ultrastructure and the composition of deposits were analyzed by TEM. VSMC were grown in MEM or DMEM in the presence of 1, 1.75, and 2mM Pi for 10 days and in the presence of 2.5 or 3.0mM Pi in MEM for 16 days (a content of more than 2mM Pi in DMEM caused massive precipitation, which is irrelevant for VC *in vivo*). Representative TEM pictures are shown in Fig 6A. VSMC grown in 1mM Pi MEM did not show relevant deposits. There were only some 50-nm nanoparticles composed of smaller particles whose structure was blurred at higher magnification. Images at low magnification (left column in Fig 6A) show that precipitates using a moderate Pi concentration (1.75mM) were quite transparent to the electron beam, which is consistent with a low Ca density and a high water content in the deposits. The density of the deposits progressively increased in 2mM content, becoming opaque in 3mM MEM. The microstructure of the deposits was revealed in images obtained at medium magnification (central column). With 1.75mM Pi in MEM, very heterogeneous deposits were observed, formed by sheets containing particles of denser material with a variety of shapes and sizes. However, when 1.75mM Pi was used in DMEM, a more organized structure was found, consisting of irregular, denser sheets that included elongated nanoparticles organized radially. Deposits in 2mM Pi MEM did not show such elongated particles, which were clearly outlined against the background in the corresponding DMEM deposits. In 2.5mM and 3mM MEM, the majority of the structure was formed by electron-dense, 10-nm long rod-like particles arranged in spherulite formations. High magnification images (right column) showed nanoparticles with diffuse contours in 1.75mM and 2mM Pi MEM deposits. In DMEM deposits with a 1.75mM Pi content, rods with a thickness of 3 nm and rounded, poorly defined 5–10 nm nanoparticles were observed, and at 2mM Pi, deposits already had straight faces, although crystal planes were not yet visible (these were only observed in the 2.5mM and 3mM MEM deposits).

The crystallinity of the deposits was also observed by electron diffraction (ED; Fig 6B). When VSMC were incubated for 10 days using 2mM Pi in MEM, ED revealed no reflections from crystal planes and only revealed amorphous deposits. Conversely, in deposits from cells incubated using 2mM Pi in DMEM, ED revealed crystallization, with rings at 2.81 and 3.52Å, meaning close to those of hydroxyapatite (2.82 and 3.44Å, respectively). Finally, in VSMC incubated in MEM with 2.5 and 3mM Pi for 16 days, ED observation of the deposits clearly showed the typical pattern of hydroxyapatite. Consequently, while bicarbonate (present in DMEM) increases the pH of culture media, it also indirectly accelerates the conversion of amorphous phosphate into crystalline hydroxyapatite, which was also observed in cells incubated with a high Pi content in MEM.

## Discussion

In this study, we have analyzed the effects due to incubating VSMC *in vitro* with high concentrations of Pi to understand the relevance to VC *in vivo*. We have observed that there is a strong relationship between Pi concentration, calcium phosphate precipitation and deposition, and cell death (Fig 1); that significant cell death starts after calcium nucleation and deposition; that cell death is proportional to the amount of calcium in deposits; and that cell death is prevented by the calcification inhibitor PPi, at any concentration of phosphate (Figs 1C, 5A and 5B). These findings therefore show that cell death during calcification is not caused by direct Pi cytotoxicity, rather it is caused by the precipitation of calcium phosphates. Other causes of cell death during calcification, such as the depletion of calcium upon precipitation (Fig 5F), were discarded. This precipitation is also necessary for the osteogene expression of surviving cells *in vitro* (Figs 1B and 4) [6]. With respect to the mechanisms, cell death can occur either after eventual cell internalization of the nucleated nanoparticles in suspension, or after deposition on the cells, or after both. Osteogene expression, however, is most likely the consequence of nanoparticle endocytosis. The reason for this difference between cell death and gene expression mechanisms is that, while nanoparticles are created by homogenous precipitation upon Pi treatment several days before osteogene expression and cell death are detected, calcium deposition at 2mM Pi occurs almost simultaneously with osteogene expression: deposition is first observed after 10 days (Fig 1D), but TNAP RNA is first overexpressed after 8 days (and activity is increased as early as 4 days) and Runx2 and Msx2 after 10 days. Due to the diversity of the relevant parameters involved in this calcification *in vitro*, each parameter will be discussed separately before giving our concluding remarks.

### Role of supersaturation

Calcium phosphate nucleation, as nanoparticles in suspension (Fig 4) is the key step during calcification *in vitro*. We have observed that most, if not all, of calcium phosphate deposits are the consequence of homogeneous precipitation in solutions, and the role of heterogeneous precipitation caused by cells or cell debris still needs to be determined. Therefore, given the importance of this precipitation in this calcification model, the process should be interpreted from a thermodynamic point of view to understand the molecular species involved and, therefore, understand the major causes and the relevance to calcification *in vivo*, if any. A relevant parameter for precipitation is supersaturation, which is the level of precipitating species in solution above the saturation point, as defined in Table 1. Supersaturation is usually calculated with respect to the crystalline species [20], which is often dicalcium phosphate dihydrate (DCPD; CaHPO_4_·2H_2_O). However, it is clear from the TEM analysis of the samples in both this experiment and previous ones [17,21] that the first phase to precipitate is amorphous calcium phosphate (ACP). Thus, the relevant value for the onset of spontaneous precipitation should be that of ACP supersaturation (which depends on ACP solubility and the ionic product of the ions forming the ACP precipitate), rather than that of HAP, as it has been argued for the *in vivo* condition [22]. While HPO_4_^2-^ is the only phosphate species in DCPD, several characterization techniques, such as ^31^P NMR [23] and X-ray diffraction, have shown that ACP [24] is mostly, if not only, composed of PO_4_^3-^, which concurs with a recent report on *calcification in vitro* that used massive calcification [25]. This is crucial for predicting precipitation at a certain pH, because the concentration of HPO_4_^2-^ barely changes in the pH range of interest (7.4–8.7), so DCPD supersaturation is not modified. However, the concentration of PO_4_^3-^ species increases by 10 fold (see Fig 3F), and therefore the supersaturation and precipitation rate of ACP will increase dramatically within this non-physiological pH range. The ACP solubility product has not been clearly established, but we can consider that the ACP ionic activity product = a_Ca_·a_PO4_^0.74^·a_H_^0.22^, and we can use an orientative K_sp_ solubility product of 2.29 x 10^-11^, as proposed [26], for T = 37°C and pH = 7.4. The supersaturation values in the precipitating media (MEM and DMEM, in addition to DMEM:F12) used in this work have been estimated for ACP and other calcium phosphate phases (Table 1) with respect to the pH values that we measured in the laminar hood and in the CO_2_ incubator. These media had variable contents of phosphate (1–3mM) and bicarbonate (MEM, 26.19mM; DMEM, 44.05mM; DMEM:F12, 14.29mM). In both MEM and DMEM, there is no supersaturation with respect to ACP at the pH of equilibrium (pH∞) between the CO_2_ of the incubator and the bicarbonate of the media (see Table 1). However, during the time required to reach this equilibrium, the alkaline pH causes supersaturation at 2 and 3mM Pi, with respect to ACP. In DMEM, the supersaturation values are considerably higher than for MEM. Note that the DCPD supersaturations in MEM and DMEM are similar and are already greater than 1 in the presence of 2mM Pi, even at the pH of gas equilibrium. Moreover, it has also been observed by TEM that the amorphous precipitates are continuously transforming by solid phase reaction. They gradually become denser and richer in Ca until they convert into crystalline HAP over time. Parallel to this densification process, the solubility of the precipitate decreases, and consequently supersaturation increases until it reaches the values corresponding to HAP. It can be seen in Table 1 that all the solutions are highly supersaturated with respect to HAP. Therefore, as the structure of the precipitate approaches that of HAP, precipitation will barely be reverted by moderate pH changes and will continue until the levels of phosphate and calcium in solution are very low, actually well below the values that caused precipitation of the ACP phase in the first place. In brief, the spontaneous precipitation of calcium phosphate in MEM and (especially) DMEM solutions can occur due to an increase in pH, even if such an increase is transitory, given that the supersaturation of the amorphous calcium phosphate found in calcification deposits increases rapidly when the pH is above 8.0. Therefore, the use of concentrated bicarbonate solutions may not be adequate for studying calcium phosphate calcifications, as in the case of MEM buffers and, especially, DMEM buffers. On the other hand, low bicarbonate, HEPES-containing media, such as the DMEM:F12 buffer, do not present this kind of problem.

### Role of pH

Commercialized culture media first start to become alkaline during preparation of the media in the hood, i.e., in open air. This is because the pH of bicarbonate solutions is highly dependent on CO_2_ pressure, as it can be inferred from the following equilibria:

$${CO}_{2}(g)↔{CO}_{2}(aq)+H_{2}O↔H_{2}{CO}_{3}↔{HCO}_{3}^{-}+H^{+}$$

H_2_CO_3_ readily decomposes into H_2_O and CO_2_ (aq), and the concentration of CO_2_ (aq) depends on the CO_2_ gas pressure. When the CO_2_ pressure is low (e.g., in open air, 0.04% approximately), soluble CO_2_ is gradually released into the atmosphere, and all the equilibria are shifted to the left, with the consequent consumption of H^+^ and basification of the medium. Thus, in bicarbonate-buffered media, such as MEM and DMEM, the final pH will mainly depend on the initial bicarbonate content and the partial pressure of CO_2_, in addition to other agents such as humidity saturation (100% in the incubator), temperature, the presence of other buffers and serum, and the metabolic activity of the cells. Therefore, MEM and DMEM cannot reach the same equilibrium, because DMEM almost doubles the concentration of bicarbonate in MEM. This is the reason why MEM is recommended for 5% CO_2_ partial pressure and DMEM for 10%, but even using MEM at 5% CO_2_, we have observed slight alkalinity (Fig 3C and 3D).

Based on the aforementioned, we decided to measure the evolution of pH in the media during the usual culture operations in the laboratory: MEM, DMEM, and DMEM:F12 were prepared at 37°C in the laminar-flow hood and were immediately placed in the incubator at the same temperature and at 5% CO_2_ partial pressure, where the pH values were measured at regular time intervals using a continuous recording pH meter (Fig 3C). In open air, the pH of DMEM:F12 was 7.56, close to the expected 7.40, while MEM pH was 8.41, and DMEM pH was 8.67. In the CO_2_ incubator, the pH slowly dropped, and it reached pH 7.40 after 1 hour, only in the case of DMEM:F12, whereas it was still 7.86 in MEM and 8.06 in DMEM after one hour. The calculation of supersaturations revealed that DMEM:F12 was already undersaturated from the start with either 1, 2, or 3mM Pi. The MEM solutions were initially undersaturated only for 1mM Pi solutions, and they dropped below saturation after 21 min. for 2mM Pi solutions and after 40 min. for 3mM Pi solutions, as it is concluded from the exponential decay experiment and the one-phase exponential equation (Fig 3C). The DMEM solutions reached saturation after only 12 min. for 1mM Pi solutions and 52 min. for 2mM Pi solutions, while at 3mM Pi, it would barely reach the threshold of saturation (pH 7.95), because it coincides with the pH at equilibrium (pH∞). In summary, variations of the CO_2_ partial pressure in bicarbonate solutions may be the cause of the pH increments observed in calcification studies that have led to the spontaneous precipitation of calcium phosphate in MEM solutions, and especially in DMEM solutions. On the other hand, pH variations in the DMEM:F12 buffer are smaller, and the pH value remains close to the desired value of 7.40.

### Homogeneous precipitation

We must emphasize the fact that the existence of supersaturation does not necessarily guarantee the onset of precipitation, because there is always an induction period and a nucleation barrier, which can be lowered in heterogeneous nucleation and in the presence of nucleation promoters, such as dead cells, structural proteins, etc. Similarly, the supersaturation needed for particle growth is quite low, so precipitation will continue in the presence of seeds even at very low supersaturation. While we have not tested this possibility, calcium carbonate could take over this role, because the dissociation of HCO_3_^-^ ion produces CO_3_^2-^ ions that in the presence of Ca^2+^ may cause the precipitation of CaCO_3_. Therefore, we used published thermodynamic data [26], and we calculated the concentration of CO_3_^2-^ and the supersaturation of CaCO_3_ in different media. We then observed that the carbonate ion increases exponentially with pH, that the supersaturation of CaCO_3_ also increases substantially with pH, and that supersaturation is higher for MEM than for DMEM. In the case of MEM, for example, the supersaturation of CaCO_3_ is 2.86 at pH 8.0, and it is 1.44 (1.89 in DMEM) at pH 7.4. Therefore, MEM buffers and, to a larger degree, DMEM buffers are already supersaturated in CaCO_3_ for a Ca^2+^ content of 1.8mM, and as in the case of calcium phosphate, supersaturation will increase rapidly with pH. Nevertheless, CaCO_3_ will only precipitate when supersaturation goes above the nucleation barrier, but when this happens, it will serve as the seed for heterogeneous calcium phosphate precipitation. The low Ca/P ratios determined by EDS in early precipitates (Fig 5B) are indirect evidence that the CaCO_3_ content is low, but this is still compatible with the role of CaCO_3_ as a precipitating seed. Regardless, the high risk of CaCO_3_ precipitation once again suggests that these bicarbonate-containing media are not recommendable for calcification studies.

The experimental results on nucleation and precipitation *in vitro* (Fig 4) coincide qualitatively with our thermodynamic analysis. Precipitation is clearly enhanced in DMEM with respect to MEM. As predicted, 1mM Pi solutions do not precipitate in the observation time frame. In 2mM Pi MEM, the calculations indicate ACP undersaturation at pH_∞_ in the CO_2_ incubator, yet for 21 minutes, as from preparation in the hood, MEM is supersaturated for ACP. Moreover, observations reveal detectable nuclei formation after a long induction time (at ≤ 24 hours without cells and at ≤ 48 hours with cells), and the nuclei remain stable in suspension after 6 days in MEM without cells. In 3mM Pi MEM solutions, with higher supersaturation values for at least 40 min. starting in the hood, nuclei form after 6 hours, and deposits are visible after 48 hours. The case of 2mM Pi in DMEM is similar to 3mM Pi in MEM, but the supersaturation of CaCO_3_ is higher, which is reflected in experiments that show reduced induction times in 2mM Pi solutions and show visible precipitation already at 24 hours (Fig 4). The effects of the type of medium and the concentration of Pi in solutions are also reflected in the crystallinity of the deposits, as shown by the electron diffraction patterns (Fig 6B). A detailed ultrastructure analysis revealed a typical process of calcium phosphate precipitation, compatible with previous observations [6,17] and summarized in Fig 6. The process initiates with 1) the formation of an electron-light laminar precipitate containing organic and inorganic (calcium, phosphate) matter with low electron density and a low Ca/P ratio. This evolves into 2) the formation of calcium phosphate nanoparticles, which are not yet crystalline (they are amorphous). Time, a high pH, and Pi lead to the formation of 3) poorly developed, rod-like nanoparticles, which arrange radially into spherulites with a Ca/P ratio that is comparable to that of hydroxyapatite (1.7). Rods seem to be formed by the stacking of rounded nanoparticles, in a process that requires time and conditions that favor precipitation. HAP nanoparticles are only formed, after some aging, by the densification of material in nanoregions and molecular reorganization into crystal planes.

### Role of cells

Given that apparent spontaneous nucleation in culture media (i.e., homogeneous precipitation) is triggered by slight increases in pH and by phosphate ion activities, with the likely help of CaCO_3_ supersaturation, the role of cells in this experimental process needs to be defined. The need for an extracellular matrix acting as a calcification promoter has been traditionally admitted, but this is not the cause of calcium phosphate nucleation, which is a cell-independent process *in vitro*. The *in vitro* model of calcification sets up a very aggressive scenario for cells, which unsuccessfully control pH and prevent precipitation, as it is concluded from the increased pH and the calcium precipitation in media without cells (Fig 4A) or with dead VSMC (Fig 3B) [6,18]. A higher deposition rate using dead cells was initially interpreted as the consequence of losing the capacity to synthesize calcification inhibitors [18], but the results presented in this work suggest that the increased pH of culture media with dead VSMC plays a predominant role (Fig 3C). The pH of a medium also rises during calcification (Fig 3B), most likely as a consequence of the increasing cell death and the progressive loss of pH control, thereby causing an increase in precipitation, more cell death, and an alkaline pH, in positive feedback. The data shown in the present work therefore suggest that the initial steps of calcification *in vitro* are spontaneous events of nucleation and growth due to transitory, alkalinity-induced supersaturations. Precipitated nanoparticles of calcium phosphates can induce *trans*differentiation and/or cell death, depending on the degree of precipitation. Phenotypic changes could be involved in heterogeneous calcification and could therefore explain the significant deposition of calcium phosphates after 12 days in VSMC incubated with 2mM Pi in MEM (Figs 1D and 2B). Calcium nucleation under these conditions is scarce, and it was only observed in this study at the moment when the medium was changed (48 hours; Fig 3B). Nevertheless, it is possible that the 100-nm nuclei that form are internalized by the cells [1,5] and, after a time interval, cause *trans*differentiation and the expression of bone-related proteins (Fig 1B). Alkaline phosphatase activity, for example, which is increased after 4 days, could favor the nucleation and deposition of calcium phosphates upon the increased hydrolysis of PPi (Fig 5C). In fact, the pharmacological inhibition of AP activity partially prevents calcification *in vitro* of VSMC induced with 3mM Pi in MEM [27].

Nevertheless, despite the participation of cells in the final steps of calcification *in vitro*, alkalinity-mediated nucleation is a necessary step in this process, as it is inferred from the fact that calcification never occurs in a low bicarbonate medium such as DMEM:F12. This medium maintains a low (physiological) pH to keep ACP undersaturated (although it would still be highly supersaturated with respect to OCP and HAP), even at 3mM Pi, and consequently DMEM:F12 prevents calcification *in vitro* even after 3 weeks of incubation with 2 or 3mM Pi (data not shown).

### Relevance to vascular calcification in vivo

Spontaneous nucleation, and therefore homogeneous precipitation, is not possible in blood, even during hyperphosphatemia. First, the total free Ca^2+^ content in blood is critically constant and barely exceeds 1.25mM, while MEM and DMEM contain 1.8mM. Second, pH is controlled and never reaches the alkalinity of media in a laminar hood. Also, the content of phosphate in severe hyperphosphatemia rarely reaches a concentration of 2.5mM, and even under these extreme conditions, blood would be undersaturated with respect to ACP (*S*_(ACP)_ = 0.53).

What, then, is the relationship to VC (if there is any), if the observed supersaturation and precipitation do not spontaneously occur *in vivo*? There are many types and causes of ectopic calcification, and when related to CKD, calcification is most prominent if it is accompanied by hyperphosphatemia [1–3]. However, the pathogenesis of CKD is very complex, and in addition to Pi, waste metabolites (uremic toxins) are also accumulated, which can moreover induce the *trans*differentiation of VSMC and calcification *in vitro* [28]. The composition of blood plasma is also much more complex than current culture media, and proteins, solutes, and calcification inhibitors all take part, in addition to a controlled pH, which is sufficient to prevent precipitation. Finally, the presence of cell types other than VSMC, structures that are critical for nucleation (such as elastin fibers), and longer nosologic times also strongly differentiate between calcifications *in vivo* and *in vitro*.

Consequently, if homogeneous precipitation does not occur *in vivo*, then vascular calcification of the arteries should only occur through cell-mediated heterogeneous precipitation. The usefulness and relevance of a model with respect to VC should be clearly defined and should use conditions limited to the *in vivo* scenario. For example, active ionic concentrations and pH should obviously be similar to what are found in uremic patients. With respect to pH, metabolic acidosis is present in most patients with CKD [29], even if blood pH is not modified as a consequence of additional buffering, but we have found alkalinity during calcification *in vitro* (Fig 3). At pH 7.4, blood is undersaturated with respect to ACP, even under severe hyperphosphatemia conditions. Consequently, unless the pH of an artery wall is locally increased, the level of PO_4_^3+^ ions would be very low, and the possibility of ACP precipitation should be negligible, contrary to what we have observed *in vitro*. With respect to calcium and phosphate concentrations, ionized calcium (Ca^2+^, 50% of total calcium) in healthy human blood ranges from 1.05–1.25mM (while MEM and DMEM contain 1.8mM CaCl_2_), and it is usually not modified or even drops with CKD. A normal range of Pi in plasma is 0.90–1.45mM, but in CKD patients 1.13mM Pi is already associated with an increased risk of death [30]; 2mM Pi is common in stages 4–5 of CKD; and 3mM Pi (9.29 mg/dL) is quite exceptional. In addition, the calcium and phosphate ion activities in blood are also substantially reduced by the high ionic strength and the formation of soluble CaHPO_4_ ion pairs, which also prevents precipitation [22]. However, we can assume that the ionic strengths (and the activity coefficients) in culture media and blood are similar, based on the high concentration of NaCl and KCl. As a conclusion, in order to mimic *in vivo* conditions, VSMC should be incubated in a calcifying medium that contains an ionic strength of I = 0.15 M, ~1.25mM calcium, ~2mM Pi, and quiescence conditions (FCS < 0.5%), plus a pH at 37°C that is correctly maintained at 7.4, in addition to specific insults that *trans*differentiate the cells, such as uremic serum.

### Concluding remarks

We must admit that, despite intense international research on the pathogenesis and therapeutics of VC over the last 15 years, progress has been less than expected, and as of yet, no efficacious therapies are available for either prevention or treatment. It is our opinion that we have been using *in vitro* models that apply very diverse experimental conditions, which are usually quite unrelated to the local environment of arteries and that have been erroneously promoting massive precipitations in a short interval. Consequently, these *in vitro* models can lead and have led to the establishment of pathogenetic proposals that can be unrelated to the VC process that occurs *in vivo*, therefore masking the correct pathways in the fight against this calamitous degenerative complication. From the results of this work, we can conclude that hyperphosphatemia considerably accelerates but does not trigger ectopic calcification, and we therefore believe that research directed at the initiating events of calcium deposition should be emphasized. Most likely, such events include alterations of the local environment and nidus formation in the artery wall, which can be caused either directly by the promotion of deposition through the expression of a calcifying matrix or pH changes, or indirectly by the local nullification of calcification inhibitors, such as PPi. In either case, one or several waste metabolites that accumulate during CKD will most likely be involved in these initiating events.

## Supporting Information

S1 Datasheet All relevant data from the figures of this manuscript are included in the Supporting Information Excel file. (XLSX)
